# De Novo Mutation of the ADAMTS13 Gene with Mesenteric Ischemia in an Infant with Congenital Thrombotic Thrombocytopenic Purpura

**DOI:** 10.1155/2021/5516863

**Published:** 2021-07-06

**Authors:** Ibrahim Alharbi, Sarah Alqarni, Wed Khayyat, Amirah Almatrafi

**Affiliations:** ^1^Department of Pediatrics, Umm Al-Qura University, Makkah, Saudi Arabia; ^2^King Fahad Armed Forces Hospital, Jeddah, Saudi Arabia; ^3^Umm Al-Qura University, Makkah, Saudi Arabia

## Abstract

**Introduction:**

Congenital thrombotic thrombocytopenic purpura (cTTP) is a rare autosomal recessive disease characterized by ADAMTS13 deficiency or a severe decrease in its activity that is caused by homozygous or combined heterozygous mutations in its encoding gene. Here, we describe a de novo genetic mutation of the ADAMTS13 gene and a rare complication of cTTP in a neonate. *Case Presentation*. A full-term baby boy developed tachypnea, bradycardia, and oxygen desaturation at 2 h of life and was shifted to the newborn intensive care unit. He was oliguric in the first 24 h of life and had one episode of epistaxis. Blood-stained urine was observed in the urine catheter, and a coffee-ground-colored fluid was drained from the orogastric tube. Histopathological analysis revealed gastric perforation with features of ischemic insult. On day 8, genetic analysis confirmed the diagnosis of autosomal recessive familial thrombotic thrombocytopenic purpura and revealed a unique homozygous deletion mutation on exon 23 of ADAMTS13: c.2883del p.(Cys962Alafs^*∗*^3).

**Conclusion:**

cTTP is a rare life-threatening autosomal recessive disease with a high mortality rate. Early detection and initiation of aggressive treatment with plasma infusion could be a life-saving strategy in such cases.

## 1. Introduction

Congenital thrombotic thrombocytopenic purpura (cTTP), which is also known as Upshaw–Schulman syndrome, is a rare life-threatening autosomal recessive disease. It is caused by mutations on a disintegrin and metalloproteinase with a thrombospondin type 1 motif member 13 (ADAMTS13). In comparison to acquired TTP, cTTP is less common and has an incidence of 1/1000,000 individuals annually worldwide [1]. cTTP is often associated with multiorgan involvement: it not only mainly involves the central nervous system but also results in myocardial and renal impairment [2]. A variety of mutations have been found to cause cTTP [3–7]. In this paper, we present a de novo genetic mutation of the ADAMTS13 gene that has never been reported before in a patient with gastric ischemia and perforation, which are unusual secondary complications of cTTP.

## 2. Case Presentation

The patient was a full-term baby boy born via normal spontaneous vaginal delivery. His birth weight was 2.718 kg, and the Apgar score was 9 and 10 at 1 min and 5 min, respectively. He was born to consanguineous parents and did not have a family history of hematological disease or similar conditions. At 2 h after delivery, he developed tachypnea, bradycardia, and oxygen desaturation, and he was shifted to the neonatal intensive care unit. The neonate had oliguria in the first 24 h of life, and he also had one episode of epistaxis. Blood-stained urine was observed in the urine catheter, and a coffee-ground-colored fluid was drained from the orogastric tube. Complete blood count performed at the age of 2 h revealed a platelet count of <8.5 (according to both automated and manual examination), a white blood cell count of 36.40 × 10^9^/L, a hemoglobin level of 21.7 g/dl, and a hematocrit value of 63.9%. Based on a consultation with a hematologist, TTP was suspected due to unexplained thrombocytopenia. A full septic workup was carried out, and based on the findings, he was intravenously administered antibiotics. The patient's lactic acid level was 3.2 mmol/L, and his bone profile was normal. Furthermore, the total bilirubin level was 348.4 *µ*mol/L, and renal function profile showed that his urea level was 8.2 mmol/L (which is higher than normal for his age) and creatinine level was 178 *µ*mol/L (which is also much higher than normal for his age).

Two days later, abdominal distention was noticed. Abdominal radiography showed the presence of a large pneumoperitoneum along with spontaneous gastric perforation. An urgent pediatric surgery consultation was requested, and total gastrectomy and feeding jejunostomy were performed. Histopathological analysis revealed gastric perforation with features of ischemic insult.

On day 8 of life, genetic analysis confirmed the diagnosis of autosomal recessive familial TTP or cTTP. A unique homozygous deletion mutation was found on exon 23 of ADAMTS13: c.2883del p.(Cys962Alafs^*∗*^3). The patient received fresh frozen plasma twice a day for eight days and then once daily for three days.

## 3. Outcome and Follow-Up

Two months later, the jejunostomy tube got spontaneously dislodged on two occasions, and the abdomen became tense. Abdomen ultrasound examination showed focally dilated small bowel loops with extensive pneumatosis in the right side of the abdomen and associated extensive portal venous gas, minimal complex free fluid, and diminished peristalsis. There were no indications of cTTP recurrence. The patient was then continued weekly infusions of Fresh Frozen Plasma (FFP) 10 ml/kg IV over 1-2 hours. These plasma infusions are meant to keep ADAMTS13 at a trough level >1% and to keep platelet count >100. However, when there is an infection, we sometimes increase FFP to 3 times per week and even once daily if platelet count dropped <100. All these measures kept the baby free of symptoms for prolonged periods of time (30–60 days sometimes).

Throughout the disease course, the patient exhibited classical signs of cTTP, for instance, developmental delay, failure to thrive, renal injury, bladder hematoma, and high blood pressure. His condition further deteriorated, and he had three episodes of sepsis, pneumonia, and severe intractable pulmonary hemorrhage. The patient eventually succumbed to these complications and died at the age of 6 months.

## 4. Discussion

The glycoprotein Von Willebrand Factor (vWF) is released by endothelial cells at the site of vascular injury, where it acts as a bridging molecule for the activation of the coagulation cascade. ADAMTS13 is an important plasma metalloproteinase that plays a role in degrading large vWF multimers under normal physiological conditions [8]. The underlying pathophysiology of cTTP is a result of ADAMTS13 deficiency or a severe decrease in its activity. Such deficiency or defects in ADAMTS13 are caused by homozygous or combined heterozygous mutations, which result in the adhesion of nondegraded and ultralarge multimers of vWF that eventually cause platelet aggregation and microangiopathic hemolytic anemia [1]. The diverse nonspecific presentation of the disease ranges from acute onset of symptoms in the neonatal period, as observed in the present case, to an asymptomatic presentation that is triggered by stressors such as surgery, pregnancy, and infection [9]. Additionally, the overlapping symptoms of cTTP, immune thrombocytopenic purpura, and hemolytic uremic syndrome sometimes result in delays in the diagnosis of cTTP [10]. cTTP should be suspected in neonates with a clinical presentation of jaundice, thrombocytopenia, and severe anemia requiring exchange blood transfusion [4]. This condition can also present with life-threatening widespread multiorgan systemic thromboses and related complications, such as stroke, renal injuries, and myocardial involvement. If medical intervention is delayed, the mortality can be as high as 90% or higher [6]. In the present case, the patient had mesenteric ischemia and perforation. The findings so far indicate that the severity of the systemic involvement varies according to the ADAMTS13 level.

Management of cTTP is controversial. There is no international consensus on the proper management of such rare cases. All the available evidence is based on anecdotal evidence, case reports, and case series. A recent literature review recommended plasma infusion over plasma exchange for the management of acute episodes in infants and children with confirmed cTTP [11]. However, there is no consensus on the best prophylactic regimen. Some papers advocate FFP infusions on a weekly basis or every 10 days to maintain ADAMTS13 at trough levels of >1–3% [12], while others recommend the use of plasma-derived factors that are rich in ADAMTS13, such as KOATE-DVI [13]. In this case report, we have described a new mutation in the ADAMTS13 gene. It will add to the database of more than 150 mutations that have been reported in the gene encoding ADAMTS13 [14].

## 5. Conclusions

cTTP is a rare life-threatening autosomal recessive disease with a high mortality rate. It is caused by a mutation in the gene encoding for the ADAMTS13 protein. In neonates presenting with early onset of severe anemia, jaundice, thrombocytopenia and/or renal dysfunction, and neurological manifestations, physicians need to consider cTTP as a potential diagnosis. Timely initiation of aggressive treatment with plasma infusion could be a life-saving strategy in such cases.

## Data Availability

The data used to reach the conclusion are all listed in the references.

## References

[1] Lotta L. A., Garagiola I., Palla R., Cairo A., Peyvandi F. (2010). ADAMTS13mutations and polymorphisms in congenital thrombotic thrombocytopenic purpura. *Human Mutation*.

[2] Brandenburg V. M., Gaertner S., Lindemann-Docter K. (2004). Underestimated complications in thrombotic thrombocytopenic purpura--haemolytic uraemic syndrome. *Nephrology Dialysis Transplantation*.

[3] Hing Z. A., Schiller T., Wu A. (2013). Multiplein silicotools predict phenotypic manifestations in congenital thrombotic thrombocytopenic purpura. *British Journal of Haematology*.

[4] Camilleri R. S., Scully M., Thomas M. (2012). A phenotype-genotype correlation of ADAMTS13 mutations in congenital thrombotic thrombocytopenic purpura patients treated in the United Kingdom. *Journal of Thrombosis and Haemostasis*.

[5] Levy G. G., Nichols W. C., Lian E. C. (2001). Mutations in a member of the ADAMTS gene family cause thrombotic thrombocytopenic purpura. *Nature*.

[6] van Dorland H. A., Taleghani M. M., Sakai K. (2019). The international hereditary thrombotic thrombocytopenic purpura registry: key findings at enrollment until 2017. *Haematologica*.

[7] Kokame K., Matsumoto M., Soejima K. (2002). Mutations and common polymorphisms in ADAMTS13 gene responsible for von Willebrand factor-cleaving protease activity. *Proceedings of the National Academy of Sciences*.

[8] Sharma D., Shastri S., Pandita A., Sharma P. (2016). Congenital thrombotic thrombocytopenic purpura: Upshaw-Schulman syndrome: a cause of neonatal death and review of literature. *The Journal of Maternal-Fetal & Neonatal Medicine*.

[9] Scully M., Hunt B. J., Benjamin S. (2012). Guidelines on the diagnosis and management of thrombotic thrombocytopenic purpura and other thrombotic microangiopathies. *British Journal of Haematology*.

[10] Klukowska A., Niewiadomska E., Budde U., Oyen F., Schneppenheim R. (2010). Difficulties in diagnosing congenital thrombotic thrombocytopenic purpura. *Journal of Pediatric Hematology/Oncology*.

[11] George J. N., Cuker A. (2017). Hereditary thrombotic thrombocytopenic purpura (TTP). *New England Journal of Medicine*.

[12] Aledort L. M., Singleton T. C., Ulsh P. J. (2017). Treatment of congenital thrombotic thrombocytopenia purpura: a new paradigm. *Journal of Pediatric Hematology/Oncology*.

[13] Naik S., Mahoney D. H. (2013). Successful treatment of congenital TTP with a novel approach using plasma-derived factor VIII. *Journal of Pediatric Hematology/Oncology*.

[14] Hou L., Du Y. (2020). Two novel mutations in ADAMTS13 in a Chinese boy with congenital thrombocytopenic purpura: a case report. *BMC Medical Genetics*.

